# Development of a Stable Process for Wire Embedding in Fused Filament Fabrication Printing Using a Geometric Correction Model

**DOI:** 10.3390/ma18010041

**Published:** 2024-12-26

**Authors:** Valentin Wilhelm Mauersberger, Fabian Ziervogel, Linda Weisheit, Lukas Boxberger, Welf-Guntram Drossel

**Affiliations:** 1 Fraunhofer Institute for Machine Tools and Forming Technology IWU, Nöthnitzer Straße 44, 01187 Dresden, Germany; fabian.ziervogel@iwu.fraunhofer.de (F.Z.); lukas.boxberger@iwu.fraunhofer.de (L.B.);; 2 Professorship for Adaptronics and Lightweight Design, Chemnitz University of Technology, 09126 Chemnitz, Germany

**Keywords:** additive manufacturing, fused filament fabrication, wire-encapsulating additive manufacturing, wire deposition, embedded electronics, conductive structures, wire integration, functionalization, composites, 3D printing

## Abstract

Using a newly developed tool head with an additional rotational axis and a wire feed, wires can be directly processed in the fused filament fabrication (FFF) process. Thus, electrical structures such as conductive paths, coils, heating elements, or sensors can be integrated into polymer parts. However, the accuracy of the wire deposition in curved sections of the print track is insufficient. To improve the wire position, a geometric correction model was set up, converted into G-code, and validated using test prints for different wire parameters. For this, a sample of printed arcs was evaluated regarding wire position and embedding quality using various visual methods. This also determined the optimal cooling time for the model. The process parameters extrusion coefficient and feed were then varied to identify optimal process parameters for a stable and at the same time efficient process. By varying the wire (copper, constantan) and polymer material (PLA, PETG), the model was checked for general validity. It was found that the position of the ø 0.2 mm wire can be improved with the correction model. Different sets of parameters can be found that enable good quality of embedding and wire position.

## 1. Introduction

For several years now, additive manufacturing (AM) has been used more and more to enable the efficient use of resources and the fast and mold-free production of highly customized products and structures. It also enables the production of components that would not be possible using any other manufacturing process. In addition to their structural advantages, AM processes also offer the possibility of integrating further, e.g., conductive elements such as wires into the components during the manufacturing process, thus functionalizing them [1]. These conductors can be used, for example, to transmit currents [2], as coils [3], as heating elements [4], or to build sensors [1]. Typically, conductors are provided on separate boards, foils, or via cables and are mounted on the base component after it has been manufactured. Using AM methods, the conductive paths can be integrated directly into the structure of the component during the manufacturing process. This reduces the parts and assembly steps, which shortens the supply chain and, therefore, has the potential to make the entire process more stable. AM of conductive tracks can be performed by applying materials and semi-finished products like filaments with conductive additives [5], pastes and inks with conductive additives [2,6,7,8], low-melting-point alloys such as solder [9], or integrated wires [4,10,11,12,13].

Due to the combination of low electrical resistance and high deformability for repeated mechanical loads, wires have superior properties compared to the processes mentioned in [2]. The properties of the wire are not changed during the printing process, as the wire is only deposited and embedded. Melting of the wire does not take place due to the relatively low temperatures required for the polymer printing process. There are two methods for depositing wires: In one method, an already-coated wire, also called prepreg, is deposited with one nozzle, similar to the CFF (Continuous Fiber Fabrication) printing process [11,12]. In the other, the wire is fed separately either through [4,14] or under the nozzle [10,13]. The separate feeding of the wire offers the possibility of applying varying amounts of coating polymer to the wire and even feeding it without polymer, which enables the design of functional elements, e.g., the winding of coils [3]. Compared to feeding through the nozzle, feeding under it allows for a greater variety of wire diameters, easier changing of wires and matrix materials, and reduces deformation of the wire due to larger bending radii during deposition. However, feeding under the nozzle requires an additional rotational axis, as the wire must be fed sideways in the direction of the print track. The additional axis leads to more complex path planning and control. At the same time, the additional axis can be used to correct the wire’s position in the print track by manipulating the direction in which the wire is fed into the track, whereas in systems where the wire is fed through the nozzle, the wire’s position can only be corrected by deflecting the entire nozzle to a larger radius. This leads to inaccuracies in the path design. While in the literature the wires were mostly integrated as a straight track [4] or in curves with large radii compared to the wire diameter [3,4], a displacement of the wires in the print track occurs especially in complex layouts with tight curves, such as those that could be used for sensors or optimized heating applications. In order to achieve optimal placement here, a correction model must be implemented in the printing process, which places the deposited wire precisely and, thus, enables more complex layouts. Two non-standard sections for the explanation of the manufacturing process (Section 2) and the developed correction model (Section 3) have been incorporated into the architecture of this paper.

## 2. Wire-Encapsulating Additive Manufacturing (WEAM)

At Fraunhofer IWU (Institute for Machine Tools and Forming Technology), a prototype system was implemented that allows for the separation of media feed. Compared to a similar system [10], our approach implements the additional rotational axis into the tool head rather than rotating the print bed. Integrating the rotation, wire, and filament feed into the tool makes it possible to use the process in regular 3D printers (in our case, an e3d toolchanger [15]) and robot systems. This system is novel in design and will be described below.

**Figure 1:**
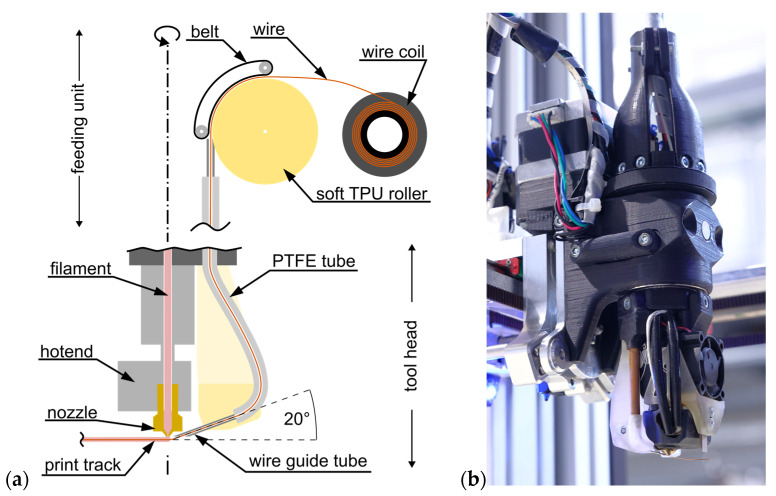
Tool head developed by Fraunhofer IWU for wire deposition processes: (**a**) Schematic diagram of the components for the material feed of polymer and wire. (**b**) Tool head mounted within the machine system.

The system consists of two main components (see Figure 1a): the tool head, which is small and light to minimize the demands on the motion system, and a feeding unit, which is positioned stationary in the machine. Both components are connected to each other via a tube system for materials and an information line. Contrary to previous approaches, the tool head is equipped with an additional axis of rotation to align the wire feed with the axis of the printing direction and, thus, optimally position the wire. The feeding unit also has a rotational axis to reduce the strain on the connecting tubes and cables. This design allows the complete system to rotate endlessly.

The developed setup is shown in detail in Figure 1. The tool head contains a hot end consisting of a heater and a nozzle for the extrusion of thermoplastics, as well as a cannula that guides the wire onto the component at an angle of 20°. Both the wire and the molten polymer are discharged in a length-controlled manner using the feeding unit. The end of the wire-feeding cannula has a 2 mm offset from the polymer nozzle, so the wire is exposed along this path. This offset is fixed in the current design of the tool head and provides sufficient spacing to the nozzle to not transfer heat while not decreasing the guiding functionality much. Furthermore, a blade is mounted on the tool head so that the deposited wire can be cut. The controlled polymer extrusion allows a selective and variable amount of embedding material to surround the wire and bond it to the base part.

Compared to other wire integration processes, the WEAM process can print larger wires at high speeds. In other studies, the processes achieved 600 mm/min [12], 900 mm/min [4], and 953 mm/min [10]. In the IWU’s predecessor process, the speed was reduced to 60 mm/min to achieve sufficient quality [12], due to position deviations in curves when driving through curves. The wire diameters used in other studies were lower, ranging from 0.05 mm [4] to 0.127 mm [10]. At our institute, the diameter of the wire used was 0.2 mm [12], and this was equal to the new WEAM process, which can deposit wires from 0.2 mm up to 1 mm.

## 3. Correction Model

### 3.1. Deviations of the Wire Position

**Figure 2:**
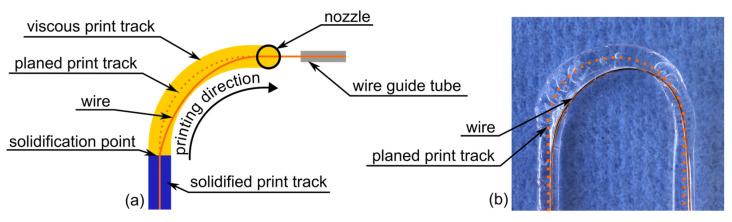
Deviation of the wire position: (**a**) Schematic representation of the wire deposition process with the resulting deviation of the wire position while printing curves. (**b**) Printed wire on a thin foil with visible deviation of the wire position.

Although the polymer nozzle itself is arranged on the rotational axis and the direction of the wire feed can therefore be changed without affecting the polymer extrusion, investigations in the past have shown that deviations of the wire position occur when printing narrow curves [10,13]. Directly after extruding the polymer, the print track has not yet completely solidified, which allows the wire to move within the print track. The motion of the nozzle while printing curves pulls the wire to the inside of the curve, as shown in Figure 2.

On the one hand, this positional deviation results in poorer quality of the wire track layout, and on the other hand, it causes the wire to become jammed in the feeding unit due to the length-controlled wire feed. To solve this problem, various correction strategies, such as limiting the print velocity, shifting the guide tube sideways, and over-/under-bending of the wire with the existing axis, have already been tested [10]. The most successful strategy was to move/bend the wire to the outside of the print track with the rotational axis to compensate for the subsequent deviation [10]. Due to its rotational axis, the WEAM print head offers the possibility to realize this deflection automatically during the printing process. In this study, a mathematical model of the behavior of the wire after leaving the nozzle was set up, which was converted into a G-code for the printing process.

### 3.2. Fundamental Idea

As mentioned in the Introduction, the position of the wire is to be improved by a correction model. For this, the following assumptions were made at first: (i) the polymer leaves the nozzle in a viscous state at a temperature above the melting point, (ii) the deposited print track solidifies after a defined cooling time, (iii) a solidification point is formed between the viscous print track and the solidified track, and (iv) the position of the wire is fixed at the solidification point.

**Figure 3:**
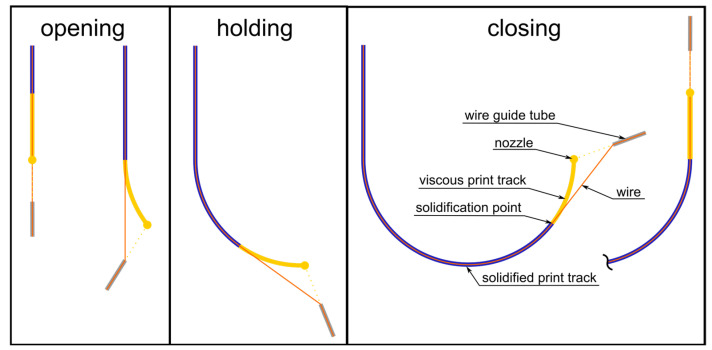
Correction model: The correction model keeps the wire tangential to the solidification point by applying an angular offset in the circular arcs. This offset is opened at the beginning of an arc, kept constant along the arc, and then closed at the end.

The fundamental idea of placing the wire in the center of the print path is to keep its position tangential to the print track at the solidification point, even in a curve. To achieve this, the alignment of the wire feed is delayed behind the direction of movement of the nozzle and adjusted to the calculated solidification point, which creates an angular offset. The movement at the circular arc is divided into the following three phases (see Figure 3):

- Opening the offset;
- Passing through the arc while holding a constant offset;
- Closing the offset at the end of the arc.

As soon as the nozzle of the tool head reaches the starting point of the circular arc, the wire feed with the cannula rotates so that the wire is held in line with the previous linear track until the solidification point reaches the beginning of the circular arc (Figure 3, opening phase). The resulting angular offset is then kept constant, and the wire feed rotates with the movement of the nozzle (Figure 3, holding phase). When the nozzle reaches the end point of the circular arc, it moves in a straight line on the following path element, while the wire feed continues to rotate and keeps the wire tangential at the solidification point, gradually reducing the angle offset (Figure 3, closing phase). As soon as the solidification point reaches the end point of the circular arc, the direction of movement of the nozzle and wire feed will be collinear again. To implement this strategy in the WEAM process, it is necessary to calculate the axis positions and convert them into a corresponding G-code to guide the movement of the printer during the three phases.

### 3.3. Mathematical Description

To transfer the presented approach for aligning the deposited wire at the solidification point, decoupled from the polymer print track, into the G-code for the printer, a mathematical description is required that clearly defines the following parameters:

- Offset angle from the wire guide tube *ε* to define the offset of the rotation of the wire guide tube.
- Distance between the wire guide tube and solidification point *a* to define the length of the wire.
- Arc length between the nozzle and solidification point *bog* to define the amount of the deposited polymer track.
- Position of the nozzle in relation to the solidification point via *ω* to describe the position of the nozzle.

**Figure 4:**
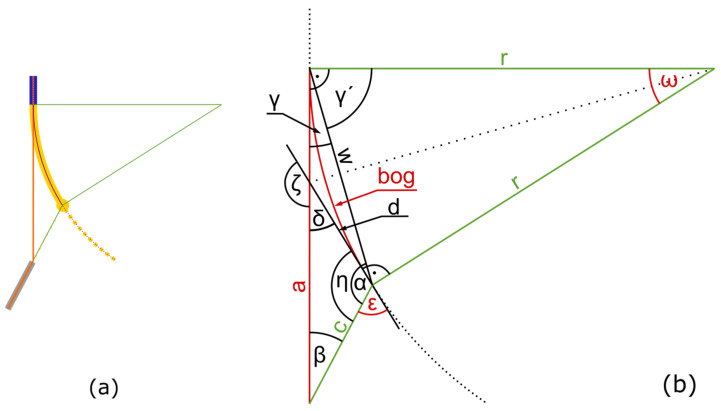
(**a**) Schematic representation of the positions of the components involved in the printing process after applying the angular offset at the beginning of the bend (opening phase of Figure 3); (**b**) representation of the geometric relationships for calculating the angular offset: known variables are shown in green, sought-after variables in red.

**Figure 5:**
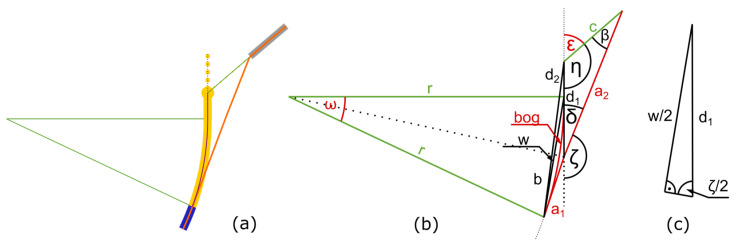
(**a**) Schematic representation of the positions of the components involved in the printing process after applying the angular offset at the end of the arc (closeing phase of Figure 3); (**b**) representation of the geometric relationships for calculating the angular offset: known variables are shown in green, sought-after variables in red; (**c**) enlarged section of (**b**).

These parameters to be calculated are shown in red in Figure 4b and Figure 5b. The following parameters are known from the printing process and can be used for the calculation:

- Distance between the nozzle and the wire guide tube *c*.
- Start, end, and center points of the arc in *x* and *y*.
- Radius of the planed print track *r*.
- Rate of movement (also called feed rate of the machine) *F*.

Another parameter required for the calculation is the cooling time *t* of the print track to reach the solidification point. However, as this is a very complex system, this parameter is dependent on many variables, such as the thermal behavior of the polymer, the wire material and diameter, and the printing speed and extrusion coefficient. Therefore, the cooling time *t* cannot be measured directly but is determined iteratively in tests, which are described in more detail in Section 4.3. In the calculations below, the cooling time *t* is used together with the feed *F* of the machine to determine the cooling distance after the nozzle to a solidification point.

The challenge of the mathematical description lies primarily in the opening and closing phases. In the opening phase at the beginning of the arc, the displacement angle between the nozzle and the wire guide tube is gradually built up according to the position of the solidification point. In the holding phase, the angle remains constant, and in the closing phase it is closed again according to the position of the solidification point until the wire guide tube and the nozzle are aligned again. However, it must be considered that the mathematical description for the opening phase differs from that for the closing phase. This is due to the different movement of the nozzle. While opening, the nozzle moves on the circular arc, and when closing, the nozzle follows a straight line.

Figure 4 shows the geometric relationships of the print track in the opening phase.

During the opening of the offset, the nozzle moves along the desired print track on a corresponding circular path with an arc length *bog* over an angle *ω* (see Figure 4). At the end of the opening phase, *bog* corresponds to the arc length between the solidification point and the nozzle *w* and can be calculated with the feed rate *F* of the machine and the required cooling time t:

(1) bog=F·t

Since *bog* is part of a circle, the angle *ω* can be calculated using the radius *r* and the arc length *bog* of this circle, which is known from Computer-Aided Design (CAD) of the print track:

$$\omega =\frac{bog}{r}$$

In combination with Equation (1), this results in the mathematical description of *ω*:

(2) $$\omega =\frac{F\cdot t}{r}$$

and since the triangle spanned by *ω* is isosceles, with sides *r*, *r*, and *w*, *a* relationship can be formulated that describes the distance between the nozzle and the solidification point *w* with only the known parameters:

(3) $$w=2\cdot r\cdot \sin\left(\frac{\omega }{2}\right)$$

According to Equation (2):

(4) $$w=2\cdot r\cdot \sin\left(\frac{r}{2\cdot F\cdot t}\right)$$

The isosceles property of the triangle also provides information about the angle *γ*′ via the geometric relationship:

$$\gamma \prime =\frac{\left(\pi -\omega \right)}{2}$$

and because the wire is held tangentially to the arc at the solidification point, the angles *γ*′ and *γ* form a right angle, which means that *γ* can be calculated as follows:

$$\gamma =\frac{\pi }{2}-\gamma \prime$$

In combination with Equation (2), this gives a clear description *γ*:

$$\gamma =\frac{\pi }{2}-\frac{\left(\pi -\omega \right)}{2}$$

(5) $$\gamma =\frac{r}{2\cdot F\cdot t}$$

The sine theorem for the triangle consisting of the sides *a*, *c*, and *w* is as follows:

$$\frac{a}{sin\left(\alpha \right)}=\frac{w}{sin\left(\beta \right)}=\frac{c}{sin\left(\gamma \right)}$$

In this triangle, the angle *γ* and the side w are known from the previous calculations. Side *c* is a familiar process parameter, as it describes the distance between the nozzle and the wire guide tube. The unknown angles *β* and *α* and the unknown side *a* can be determined as follows:

$$\beta =arcsin\left(\frac{w}{c}\cdot sin\left(\gamma \right)\right)$$

$$a=\frac{sin\left(\alpha \right)}{sin\left(\gamma \right)}\cdot c$$

and using Equations (4) and (5) results in

(6) $$\beta =arcsin\left(\frac{2\cdot r\cdot \sin\left(\frac{r}{2\cdot F\cdot t}\right)}{c}\cdot \sin\left(\frac{r}{2\cdot F\cdot t}\right)\right)$$

Using the interior angles *α*, *β*, and *γ* as well as Equations (5) and (6), the following results:

$$a=\frac{sin\left(\pi -\beta -\gamma \right)}{sin\left(\gamma \right)}\cdot c$$

(7) $$a=\frac{sin\left(\pi -arcsin\left(\frac{2\cdot r\cdot \sin\left(\frac{r}{2\cdot F\cdot t}\right)}{c}\cdot \sin\left(\frac{r}{2\cdot F\cdot t}\right)\right)-\frac{r}{2\cdot F\cdot t}\right)}{sin\left(\frac{r}{2\cdot F\cdot t}\right)}\cdot c$$

This provides a clear mathematical description of the second required parameter *a* (distance between the wire feed tube and solidification point for the wire length).

As the lines *a* and *d* are tangential to the arc, the straight line between their point of intersection and the center of the circle divides the angles *ω* and *ζ* equally. A triangle can be formed from the intersection of the lines *a* and *d*, the starting point of the arc, and the center of the circle. The interior angle is calculated as follows:

$$\pi =\frac{\omega }{2}+\frac{\zeta }{2}+\frac{\pi }{2}$$

therefore,

(8) ζ=π−ω

The secondary angle *δ* can be calculated as follows:

δ=π−ζ

and using Equation (8) results in

$$\delta =\pi -\left(\pi -\omega \right)$$

(9) δ=ω

Using Equation (2) results in

(10) $$\delta =\frac{F\cdot t}{r}$$

To calculate *ε*, the opposite angle *η* can be used, which can be determined by the interior angle with *β* (Equation (6)) and *δ* (Equation (10)), resulting in

ε=π−η

(11) η=π−β−δ

ε=π−(π−β−δ)

(12) ε=β+δ

(13) $$\varepsilon =arcsin\left(\frac{2\cdot r\cdot \sin\left(\frac{\omega }{2}\right)}{c}\cdot sin\left(\frac{\omega }{2}\right)\right)+\frac{F\cdot t}{r}$$

With Equation (13) (offset angle *ε*) and Equation (7) (wire length *a*), all parameters for the opening phase are known. An interpolation calculates multiple points during the opening phase to create G-code lines. An example of this G-code is shown in Appendix B.

During the holding phase, the calculated offset angle *ε* is kept constant. For the closing phase at the end of the arc, another calculation must be carried out to determine the required parameters. Figure 5 shows the associated description.

During this phase, the nozzle already moves in a straight line again, while the wire must still be guided in an arc. Therefore, the print track used for the calculations is divided into an arc section *bog* and a straight section *d*_2_. The ratio of this split changes as the solidification point approaches the end of the arc. The closing phase starts when the nozzle is at the end of the arc. At this point, all of the print track is still in the arc, so Equations (1) and (2) are still valid and can be used to calculate this point. After that, the split into *bog* and *d*_2_ is transferred more and more into the straight line *d*_2_, and the remaining arc with its length *bog* decreases.

$$F\cdot t=d_{2}+bog$$

$$d_{2}=F\cdot t-bog$$

(14) $$d_{2}=F\cdot t-\omega \cdot r$$

Equations (3), (8), (9), (11) and (12) remain valid. Equations (6) and (7), and with them the calculations for the offset angle *ε* and wire length *a*, change due to the split into the arc and straight line. A new sine theorem for the calculation of *β* and *a*_2_ can be formed from the lines *a*_2_, *d*, and *c*:

$$\frac{a_{2}}{sin\left(\eta \right)}=\frac{d}{sin\left(\beta \right)}=\frac{c}{sin\left(\delta \right)}$$

(15) $$\beta =arcsin\left(\frac{d}{c}\cdot sin\left(\delta \right)\right)$$

(16) $$a_{2}=\frac{sin\left(\eta \right)}{sin\left(\delta \right)}\cdot c$$

The distances *a* and *d* are split up, and the following applies:

(17) $$a=a_{1}+a_{2}$$

(18) $$d=d_{1}+d_{2}$$

(19) $$a_{1}=d_{1}$$

The only unknown length in Equation (15) is *d*, which can be calculated with Equation (18). One part of length *d* is known due to Equation (14), and the missing *d*_2_ can be obtained by the sine theorem of the triangle shown in Figure 5c:

$$d_{1}=\frac{w}{2\cdot sin\left(\frac{\zeta }{2}\right)}$$

In combination with Equations (3) and (8), this results in

(20) $$d_{1}=\frac{r\cdot sin\left(\frac{\omega }{2}\right)}{cos\left(-\frac{\omega }{2}\right)}$$

$$d=\frac{r\cdot sin\left(\frac{\omega }{2}\right)}{cos\left(-\frac{\omega }{2}\right)}+F\cdot t-\omega \cdot r$$

This can be combined with Equations (9) and (15) to obtain *β*, as well as the offset angle *ε* with Equation (12).

(21) $$\beta =arcsin\left(\frac{\frac{r\cdot sin\left(\frac{\omega }{2}\right)}{cos\left(-\frac{\omega }{2}\right)}+F\cdot t-\omega \cdot r}{c}\cdot sin\left(\omega \right)\right)$$

(22) $$\varepsilon =arcsin\left(\frac{\frac{r\cdot sin\left(\frac{\omega }{2}\right)}{cos\left(-\frac{\omega }{2}\right)}+F\cdot t-\omega \cdot r}{c}\cdot sin\left(\omega \right)\right)+\omega$$

Finally, the wire length *a* is calculated with Equation (17). The first part of length *a* can be found by using Equation (20) via the relationship of *a*_1_ and *d*_1_ in Equation (19). The second part can be by Equation (16) and its inputs Equations (9), (11) and (21).

$$a_{1}=\frac{r\cdot sin\left(\frac{\omega }{2}\right)}{cos\left(-\frac{\omega }{2}\right)}$$

$$a_{2}=\frac{sin\left(\pi -\beta -\omega \right)}{sin\left(\omega \right)}\cdot c$$

$$a_{2}=\frac{sin\left(\pi -arcsin\left(\frac{\frac{r\cdot sin\left(\frac{\omega }{2}\right)}{cos\left(-\frac{\omega }{2}\right)}+F\cdot t-\omega \cdot r}{c}\cdot sin\left(\omega \right)\right)-\omega \right)}{sin\left(\omega \right)}\cdot c$$

(23) $$a=\frac{r\cdot sin\left(\frac{\omega }{2}\right)}{cos\left(-\frac{\omega }{2}\right)}+\frac{sin\left(\pi -arcsin\left(\frac{\frac{r\cdot sin\left(\frac{\omega }{2}\right)}{cos\left(-\frac{\omega }{2}\right)}+F\cdot t-\omega \cdot r}{c}\cdot sin\left(\omega \right)\right)-\omega \right)}{sin\left(\omega \right)}\cdot c$$

With Equation (23) (offset angle) and Equation (22) (wire length), all parameters for the closing phase are known. As in the opening phase, an interpolation calculates multiple points to create G-code lines. An example of this G-code is shown in Appendix B.

## 4. Materials and Methods

### 4.1. Materials

In this research, print tracks made of wire and polymer were applied to base parts. Here, the material of the base part was selected to that it matched the polymer of the print track to guarantee optimal adhesion. Due to their good processing properties and wide range of applications, PLA (polylactide) [16] and PETG (polyethylene terephthalate glycol) [17] are currently the most popular polymers in 3D printing. Because the WEAM print tracks should be integrated into regular 3D-printed objects, these materials were investigated in this study. The used PLA was manufactured by Raise3D, Irvine, CA, USA, the PETG by FILAMENTWORLD, Neu-Ulm, Germany. They also differ in their crystalline structure—PLA is a semi-crystalline polymer and PETG is an amorphous polymer [18,19]—and, thus, in their processing properties. For the later use of CT (computed tomography) imaging to examine the position of the wire in the print track, an additional layer of copper-particle-filled PLA from Formfutura, Nijmegen, The Netherlands [20] was applied to the base parts. This provided contrast to the wire. The wires were selected due to their possible applications, as shown in Table 1. The WEAM tool head is currently limited on the low end to ø 0.2 mm wire due to buckling of smaller wires. Especially for sensor structures like strain gauges, even thinner constantan would be preferable but could not be tested. All tested wires where manufactured by BLOCK, Verden, Germany.

### 4.2. Test Samples

**Figure 6:**
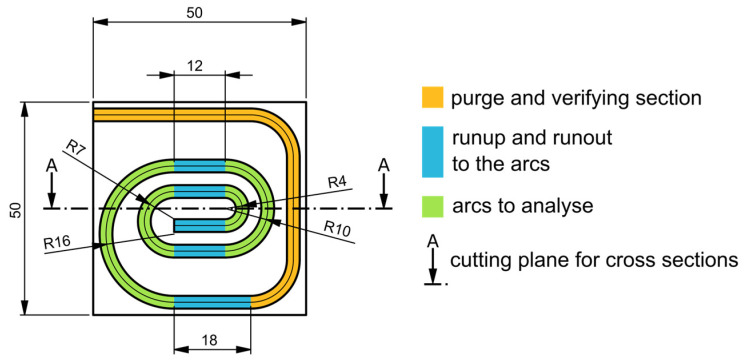
Specimen geometry.

To validate the correction model as well as to subsequently optimize the process parameters, various test prints must be carried out in which the quality of the print can be evaluated based on the position of the wire and its embedding. Therefore, a new specimen geometry, as shown in Figure 6, was constructed. A 3 mm wide track was provided on the specimen for depositing the wire. The track started with a purge and verification section (yellow part in Figure 6) and was followed by a test section consisting of four circular arcs with decreasing radii (green parts in Figure 6), as well as a straight runup and runout to the arcs (blue parts in Figure 6). On the purge and verification section, the polymer flow and wire feed can stabilize. The calibration of the tool head was also verified on this section. The position of the wire was measured on the runup and runout to the arcs, as well as in the arcs themselves. The embedding of the wire into the polymer was evaluated only in the arcs.

On these test samples the following parameters were varied: wire diameter and material, embedding material (polymer), extrusion coefficient, and feed. The wire diameter and material provide information about the influence of the thermal metal properties on the embedding process and influence the mechanical behavior of the wire during the printing process. The extrusion coefficient describes the ratio of extruded filament to deposited wire and, hence, defines the amount of polymer embedding the wire. For a coefficient of 0.1, if 10 mm of wire is deposited, 1 mm of ø 1.75 mm filament is pushed into the nozzle.

The samples were printed with a feed of 1000 mm/min, a nozzle distance of 1 mm, and a hot-end temperature of 225 °C. The feed was a standard speed, and the nozzle distance was determined in preliminary tests.

To validate the correction model, printing tests were carried out in which the print bed was replaced by a glass plate with a camera positioned underneath. First, a 0.1 mm film of the embedding material was printed onto the glass to ensure optimal adhesion of the print track. A print track with integrated wire was then deposited in arcs, as described above. To detect the behavior of the wires after leaving the guide tube, several images were taken.

### 4.3. Experimental Determination of Cooling Time

To determine the correct cooling time, printing tests on a copper wire with a diameter of 0.2 mm in PLA were carried out, in which the cooling time t in the correction model was successively reduced from 2.0 s to 0.25 s. The printed results were evaluated regarding the embedding grade and position via the standard deviation and arithmetic mean of the wire, and the optimal cooling time, with a good balance between an accurate position and acceptable embedding, was derived from the results.

### 4.4. Determination of the Embedding Quality

**Figure 7:**
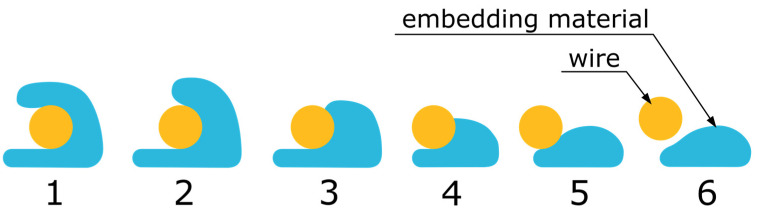
The grading system used to evaluate the embedding quality of the wire in the surrounding polymer.

The embedding quality describes how completely the wire is surrounded by the polymer. Since the embedding of the wire on straight lines is always complete, only circular arcs were examined here. Unlike measuring the wire position, the embedding was evaluated using a grading system. The sorting into six clearly defined criteria enables an assessment to be made by looking at the samples. The criteria are explained below, as well as in Table 2, and are shown in Figure 7. The best possible embedding (grade 1) is achieved when the wire is embedded at least in a u shape and is covered by the polymer. Grade 2 is awarded when the wire is enclosed but not completely covered by the polymer. If the wire is not covered, a score of 3 is given if the top end of the polymer is above the wire, and a score of 4 is given if the wire is above the polymer. A score of 5 is given if the wire is not covered by the polymer but is resting on it. A score of 6 is given if the wire has already become detached.

**Figure 8:**
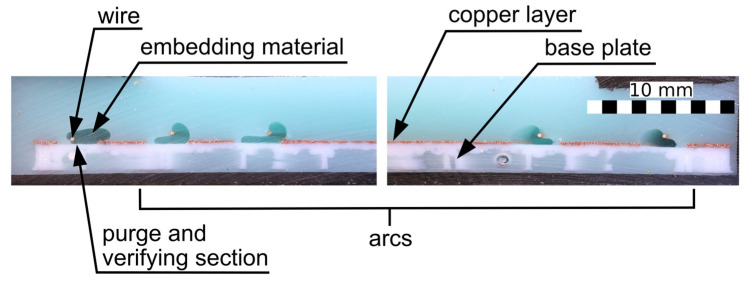
Cross-sectional image of a sample. These images were used to verify the grading system.

To validate this system, cross-sections of prominent specimens were prepared by cutting, as shown in Figure 6, and captured using a camera (Canon EOS R6, Tokyo, Japan) with a macro lens (100 mm f2.8l macro is usm). One such section can be seen in Figure 8.

All 4 arcs were evaluated, and an average value was calculated from the ratings.

### 4.5. Determination of the Wire Position

**Figure 9:**
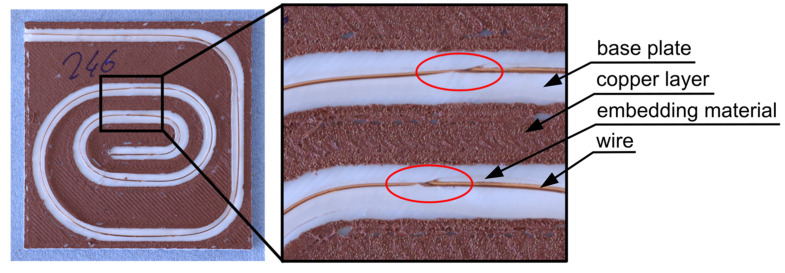
Test sample consisting of a base plate, copper layer, copper wire, and the polymer for embedding the wire. The oval shape of the print track causes optical errors when viewing the wire.

The wire position in the print track could not be determined using visual inspections or a microscope due to optical lens effects skewing the wire position in the transparent embedding polymer (see red markers in Figure 9), or due to the wire not being visible at all in the opaque polymer. Therefore, computed tomography (CT) was used as an imaging method to obtain a 2D view of the wire in the specimen. The images were taken with an X-ray inspection system (V|tome|x S 240 by GE Sensing & Inspection Technologies, Wunstorf, Germany) with 120 kV voltage, 200 µA current, and an exposure time of 333 ms. To achieve sufficient contrast to the wire, a layer of copper-filled filament was printed onto the test specimens.

**Figure 10:**
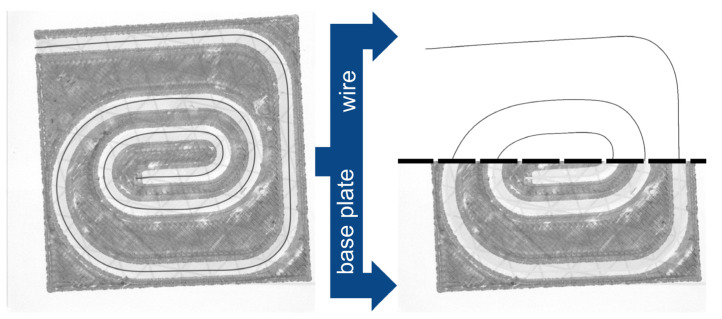
Separation of the CT image: The CT image (**left**) is separated, as shown by the black dashed line, into the wire (**right top**) and the base plate (**right bottom**) based on the color value of the pixels.

**Figure 11:**
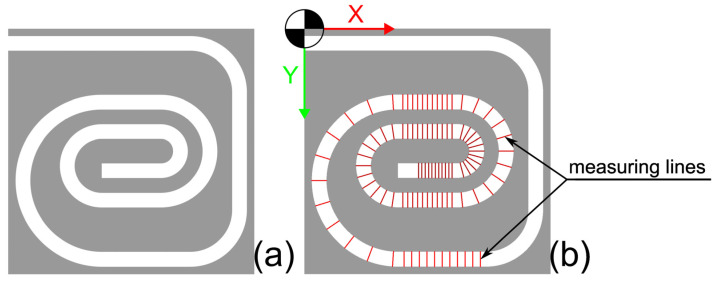
Alignment mask and measurement map: (**a**) The base plate from Figure 10 was aligned using a mask of the copper layer. (**b**) The aligned wire was measured using the red lines on the measuring map.

**Figure 12:**
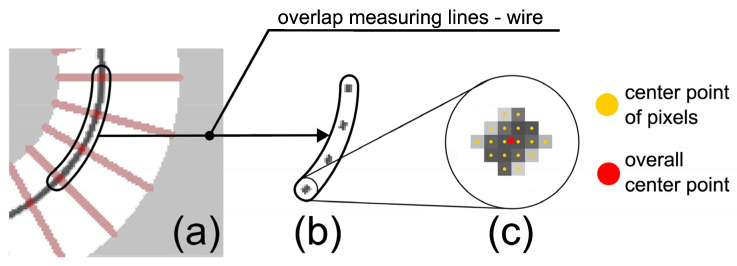
Measuring method: (**a**) To measure the wire, it was overlaid with the measurement lines of the measurement map from Figure 11. (**b**) The pixels of each intersection serve as one measuring point each. (**c**) Within the measuring points, the center point is formed.

**Figure 13:**
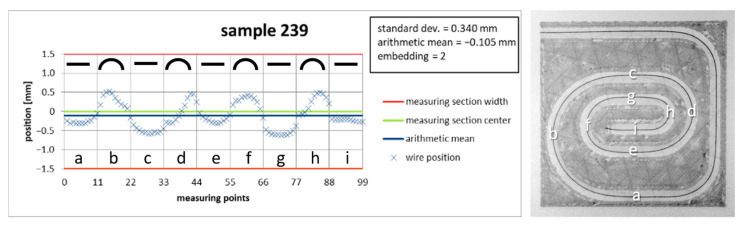
Diagram of the wire position along the print track, with values for standard deviation and arithmetic mean, as well as the quality of the embedding described in the previous section. Each segment of the print track has 11 measured wire positions. The mapping of the segments to the parts is shown by a–i.

For the consistent evaluation of the images resulting from the CT scans, a program was developed and programmed in Python 3.10 with cv2 4.6.0.66, which performed the following steps:

1. Separation of base part and wire based on the color value (the wire has a darker shade of gray compared to the thin copper filament layer) (Figure 10).
2. Aligning the base part to a mask from CAD (Figure 11), resulting in a displacement matrix.
3. Aligning the wire according to the displacement matrix obtained from Step 2.
4. Extraction of points from the wire using measurement lines (Figure 11 and Figure 12).
5. Calculation of the deviation of the measured points from the planned wire position.
6. Generation of a diagram of the wire position, as well as calculation of the characteristic values: standard deviation and mean value (Figure 13).

Using this method, all parameters relevant for evaluating the position of the wire can be determined automatically. The arithmetic mean provides information about the centering of the wire in the print track. The closer it is to zero, the more centered the wire is. The standard deviation provides information about the extent to which the wire deflects from the average position.

## 5. Results

Using the methods described above, the geometric assumptions of the correction model were first validated. The extrusion coefficient was then varied to identify an optimum. In addition, parameter studies were carried out with different wire and polymer materials to check the general validity of the correction model.

### 5.1. Validation of the Model

**Figure 14:**
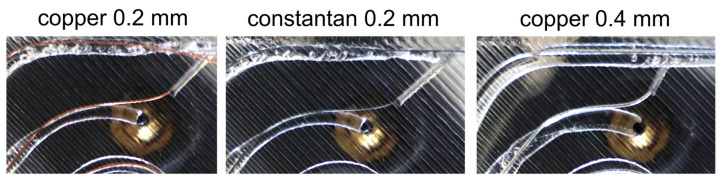
Pictures of the embedding process with the correction model. The images show the behavior of the wire after leaving the guide tube.

To validate the correction model, images such as those shown in Figure 14 were taken during the holding phase (Figure 3) when passing through an arc to check whether the geometric assumptions that we made corresponded to reality.

**Figure 15:**
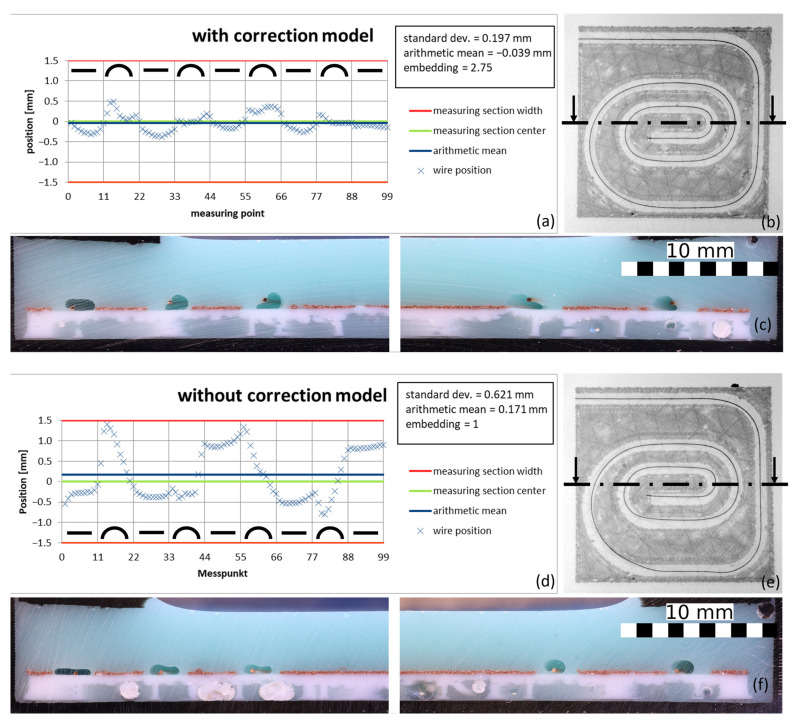
Comparison of the wire position with and without the correction model in diagrams (**a**,**d**), as well as the associated CT-scans (**b**,**e**), with the cutting planes drawn in for cross-sections (**c**,**f**). (Feed 1000 mm/s, PLA with extrusion coefficient of 0.4, 0.2 mm copper wire, and 1 mm nozzle height for both samples, with a cooling time of 0.25 s for the correction model).

It was found that the geometric assumptions made in the model are valid for prints with a wire diameter of 0.2 mm. However, a diameter of 0.6 mm could not be printed, and the 0.4 mm wire had larger bending radii, resulting in deviations from the model. Thicker wires have a higher bending stiffness and, therefore, form larger bending radii. Due to the correction model assuming a straight line from the end of the guide tube to the solidification point, high bending radii result in deviations from the calculations and, thus, deviations in the wire length at the nozzle position. This causes the wire to be pulled too far into the print track. An evaluation showed that the positional accuracy with the correction model for the 0.4 mm wire was worse than without the model. The even higher deviations of the 0.6 mm wire resulted in it being pushed out of the print track entirely and, thus, not being attached at all. Therefore, further tests to optimize the process parameters were carried out on a copper wire with a diameter of 0.2 mm. To reduce the effects of the larger bending radii, the print head could be adapted so that the distance between the nozzle and the wire guide tube is large enough to ensure that larger minimum bending radii of the wires do not push them out of the polymer track when they are deposited. Alternatively, the correction model could be extended to include dependence of the bending radii on the wire diameter. Figure 15 shows a comparison of an embedded copper wire with and without the correction model with the same set of parameters, using the position diagrams, the evaluation variables mean value, standard deviation, and embedding, and the evaluated cross-sections.

The diagram shows that the wire’s position curve is significantly smoother with the correction model. This is also reflected in the reduced standard deviation of 0.197 mm with the correction model, compared to 0.621 mm without. Without the model, the wire is not in the middle of the print track—the arithmetic mean of 0.171 mm is significantly above zero. With the model, the mean value shifts into the negative (−0.039 mm) but is clearly closer to zero. Only the embedding is better without the model than with it. The reason for this is that the wire always lies directly in the print track and is therefore always encapsulated, whereas in the process with a correction model it is always pulled sideways into the polymer track in the arcs. The challenge now lies in optimizing the process parameters to achieve sufficiently good embedding even with the model.

### 5.2. Cooling Time

**Figure 16:**
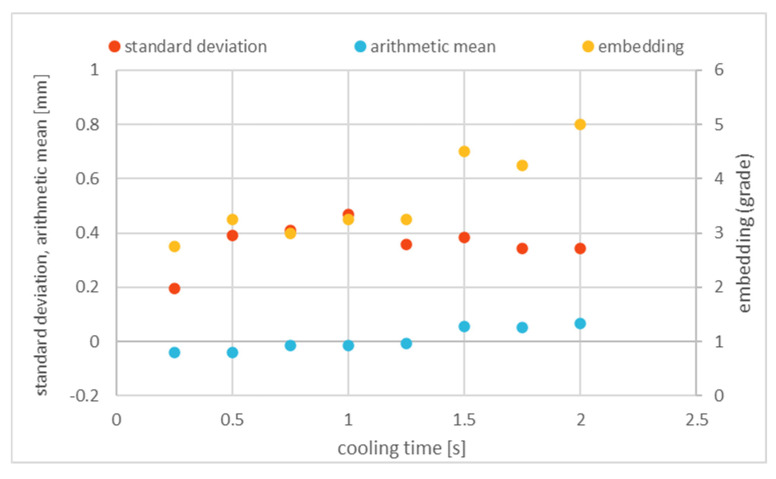
Dependence of the print quality on the cooling time *t* of a copper wire with a diameter of 0.2 mm in PLA with a feed *F* = 1000 mm/min and extrusion coefficient *c* = 0.4 mm.

With all of the selected cooling times in the correction model, it was possible to print evaluable samples. Figure 16 shows a representative result in the form of a test series with copper wire with a diameter of 0.2 mm in PLA, with a feed of 1000 mm/min and an extrusion coefficient of 0.4.

The results show that reducing the cooling time has only a marginal effect on the arithmetic mean. This is shifted from 0.07 mm at 2 s cooling time to −0.04 mm at 0.25 s cooling time and lies well in the middle of the print track. However, the embedding quality shows a clear improvement when the cooling time is reduced. Thus, the embedding quality improves from 5 to 2.5, which means that at a cooling time of 2 s the wire is not embedded with polymer, but at 0.25 s it is at least partially embedded. Similar correlations can be found for the other parameter combinations in Appendix A.

If longer cooling times are assumed in the correction model, the print track is already slightly cooled when the wire is fed in; therefore, it has a higher viscosity, which means that the wire cannot penetrate as far into the print track and lies on the outside of the print track (the arithmetic mean is above zero in the positive range). This position leads to poor embedding but stabilizes the position of the wire, resulting in a low standard deviation. By reducing the cooling time, the wire penetrates deeper into the embedding polymer, which leads to greater freedom of movement of the wire. Although the free movement leads to a higher standard deviation, the deeper penetration improves the embedding and the mean deviation decreases. With a cooling time of 0.25 s, a value was found for the correction model that achieves good results. To further increase the print efficiency, additional steps could be taken to physically reduce the cooling time even further. There are two options for reducing the cooling time: On the one hand, the heat energy supplied can be reduced by using an even lower amount of embedding polymer. This would be an option for thinner wires and could be used with even higher feeds with the current melting performance. On the other hand, the use of a compressed air nozzle in the vicinity of the nozzle could be an option to reduce the cooling time but not increase the feed with the current hot end. However, due to the need for rotational decoupling, the supply of compressed air is complex.

### 5.3. Process Optimization

**Figure 17:**
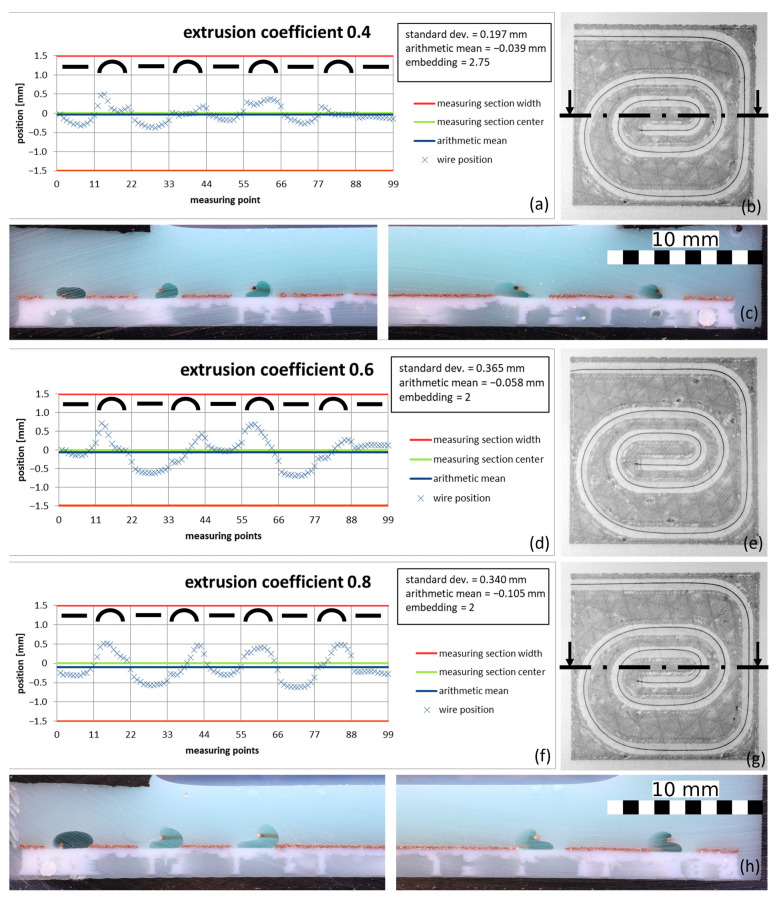
Wire positions for different extrusion coefficients. Measured position along the wire in diagrams (**a**,**d**,**f**), as well as the corresponding CT images (**b**,**e**,**g**), with the cutting planes drawn in for cross-sections (**c**,**h**) (feed 1000 mm/s, PLA polymer, 0.2 mm copper wire, height of the nozzle 1 mm, and cooling time of 0.25 s for the correction model for all samples).

After validating the model and identifying the optimal cooling time for a 0.2 mm copper wire in PLA, the process parameters extrusion coefficient and feed were specifically varied to identify an optimum that not only delivers good-quality results but also makes the process more effective. The extrusion coefficient was chosen as the parameter to be varied, as the amount of polymer applied has an influence on the cooling behavior and, thus, on the quality and speed of the process. On the other hand, there is the potential to make the process more resource-efficient by saving material. The feed parameter has a direct and significant influence on the speed of printing and, therefore, on the time efficiency of the process. Figure 17 shows the position diagrams of a 0.2 mm copper wire in PLA with different extrusion coefficients.

The embedding becomes slightly worse as the extrusion coefficient decreases. On the other hand, both the arithmetic mean and the standard deviation decrease. This means that the position of the wire in the print track improves as the extrusion coefficient decreases. In terms of resource efficiency, a subsequent increase in printing speed to enhance efficiency, and possible wire compaction in coils, a lower extrusion factor is preferable, as less embedding material is required. This also has a positive effect on the cooling time t. In addition, a lower factor generally produces a thinner print track, making more filigree patterns possible. Therefore, an extrusion factor of 0.4 was chosen for the further tests.

In order to increase the efficiency of the process, the feed was increased in subsequent tests up to 3000 mm/min. Due to the limited melting capacity of the hot end (35 mm^3^/s under a best-case scenario [21]; with a 0.4 extrusion coefficient, this would be a theoretical maximum of 2182 mm/min.), the extrusion coefficient had to be lowered further to 0.2. This ensures that no operation takes place at or over the performance of the hot end, and with this a constant extrusion of the polymer. The reduction in the amount of coating polymer is accompanied by a reduction in the cooling time to 0.075 s. In the new parameter space with a thinner print track, samples were produced that showed that the height of the nozzle must be reduced to 0.6 mm for the thinner print track to achieve a similar embedding (grade 5.5 for height 1 mm, grade 3.5 for height 0.8 mm, grade 2.5 for height 0.6 mm) as with a larger track. This second parameter set provides an alternative set of process parameters for rapid printing. However, this set of parameters requires a precise height above the part surface due to the small print track and, thus, is not applicable to all applications.

### 5.4. Material Variation

Alternative materials were examined to investigate the general validity of the correction model. Therefore, constantan was chosen as an alternative wire material, as this is a common material for the production of sensors or heaters, but with a Young’s modulus *E* = 180 GPa, a thermal conductivity *λ* = 49 W/mK, and a specific heat capacity *c* = 410 J/(kg K), it has different mechanical and thermal properties than copper (*E* = 115 GPa, *λ* = 380 W/mK, *c* = 383 (J/kg K)).

**Figure 18:**
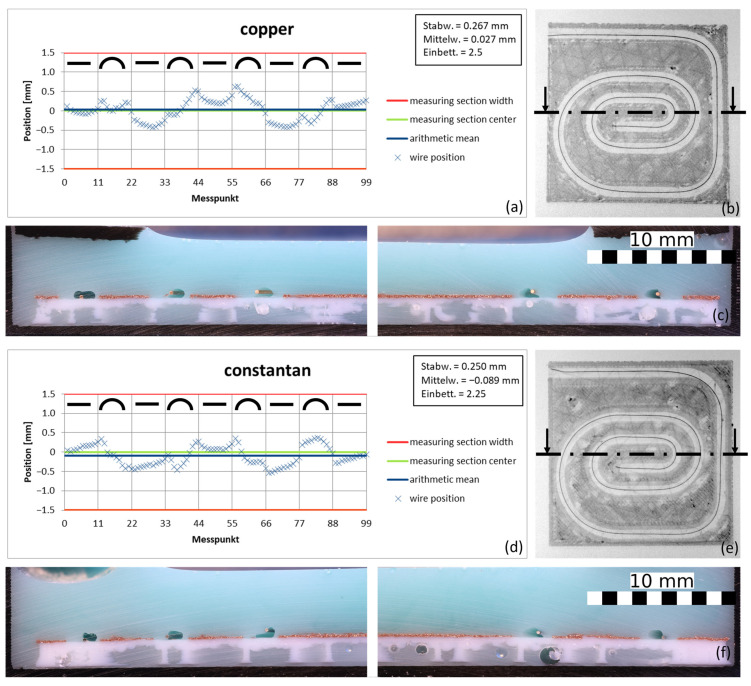
Comparison between the behavior of copper and constantan wire at the same printing parameters: Measured wire positions in (**a**,**d**), as well as the associated CT scans (**b**,**e**), with the cutting planes drawn in for cross-sections (**c**,**f**) (feed 3000 mm/s, PLA polymer, 0.2 mm copper wire, height of the nozzle 0.6 mm, and cooling time of 0.075 s for the correction model for all samples).

Figure 18 shows the position diagrams of 0.2 mm copper and constantan wires printed in PLA, as well as their embedding evaluation.

Both the position and the embedding of the compared wires were very similar. This means that both wires can be processed with the correction model in the WEAM process, which indicates a certain general validity with respect to the wires used for this process variant.

**Figure 19:**
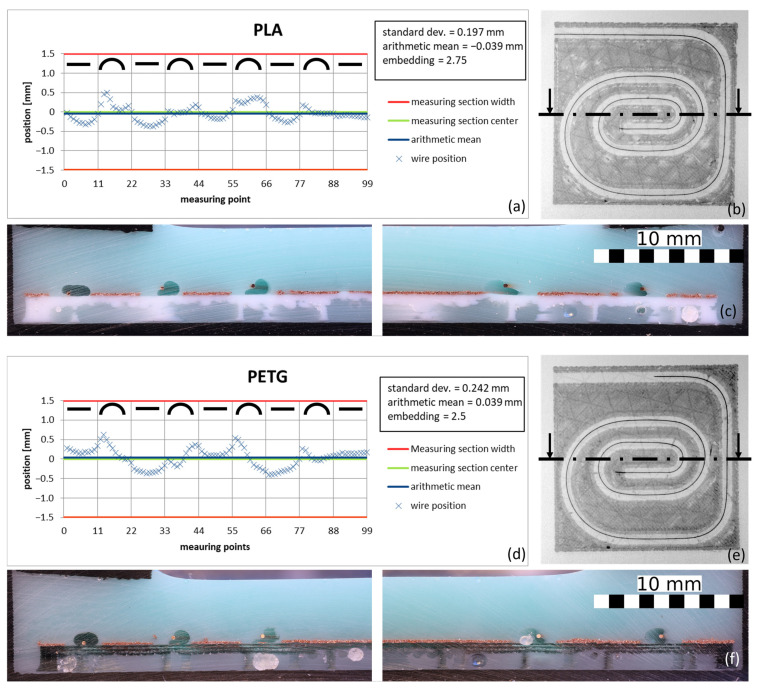
Wire positions for different embedding materials with a copper wire with a diameter of 0.2 mm. Measured wire position (**a**,**d**), as well as the associated CT scans (**b**,**e**), with the cutting planes drawn in for cross-sections (**c**,**f**) (feed 1000 mm/s, PLA polymer, 0.2 mm copper wire, height of the nozzle 1 mm, and cooling time of 0.25 s).

PETG, a polymer commonly used in 3D printing, was chosen as an alternative embedding material. It has slightly different characteristic temperatures then PLA. Both the melting point Ts at 200–230 °C and the glass transition temperature T_G_ at 80 °C are higher than for PLA (T_G_ = 60 °C, T_S_ = 160 °C). Figure 19 shows the position diagrams in a comparison of prints with PLA and PETG. Both polymers were printed with a hot-end temperature of 225 °C.

Although PETG with 2.5 shows better embedding than PLA with 2.75, the standard deviation of 0.242 mm is higher than that of PLA, with 0.197 mm. However, the differences between the two polymers are not that great, which means that a general validity can also be concluded for the embedding material used, provided that the characteristic temperatures of the polymer are within similar limits.

## 6. Discussion

Two sets of parameters were identified for a stable process (see Table 3). While parameter set A with a feed of 1000 mm/min focuses on the highest print quality and can also be used on slightly uneven surfaces thanks to the nozzle height of 1 mm, parameter set B provides a process that is primarily designed for efficiency with a feed of 3000 mm/min. However, a very even surface is essential here due to the reduced nozzle height.

Within the identified parameter sets, it is now possible to produce more complex structures with correspondingly small bending radii at high process speeds and still achieve good quality. The achievable positional accuracy is particularly relevant for the aforementioned coils and antenna applications. The high processing speeds enable the process to be used economically as a replacement for the cable structures or circuit boards that have been used up to now. Due to its increased accuracy, this process can be used in smaller structures that are typically reserved for processes involving the deposition of liquid or viscous conductor materials (see Introduction). Manufacturing these with the better electrical properties of conventionally manufactured wires (compared to liquid or viscous pastes) makes the process suitable for small sensors. The process thus advances the trend of functionalizing normally passive additively manufactured parts.

The need for high-quality wire integration with a position-enhanced WEAM process can be seen in a wide range of industries. Most prominent are the following:

- In the automotive sector, due to its high standards as a replacement for conventional cable harnesses to reduce assembly costs.
- In the aviation and aerospace industry, for the integration of accurate sensor structures into the base components to reduce weight.
- In consumer electronics, for efficient integrated antenna coils for cost-effective on-demand production

In subsequent research, the thermal influence of the wire on the cooling and solidification behavior is to be investigated in more detail using simulation methods. Using these simulations, the cooling time determined iteratively in this work can be determined deterministically and, thus, predicted for different material combinations. Due to the focus of this method on electrical integration, subsequent work will continue to examine metallic wires in coils or meander-like structures. In addition, the validity of the correction approach must be confirmed on three-dimensional surfaces.

## 7. Conclusions

The investigation showed that it is possible to significantly improve the position of the wire in the print track when passing through arcs using a geometric correction model and the print head’s axis of rotation integrated in the WEAM process, thus creating a stable process for wire embedding. However, the improved results could only be achieved for wires with a diameter of 0.2 mm. To achieve validity of the model for a wider range of wire diameters and materials—and, with that, the possibility to print more complex geometries—the correction model should be extended further. Most importantly, the bending radii of the wires after leaving the guide tube should be calculated and included depending on the diameter and material. Furthermore, the effects of the thermal behavior of different wires on the cooling behavior, as well as the external cooling and external conditions, must be investigated in subsequent work.

It was also shown that, while a higher extrusion coefficient slightly improves positioning and embedding, a lower extrusion coefficient achieves sufficiently good results. Therefore, an extrusion coefficient of 0.4 was chosen as optimal for a feed of 1000 mm/min in terms of resource and process efficiency and functionality. The variation of both the wire and the embedding material showed only a slight influence on the embedding quality and the position of the wire, which suggests that this model is generally valid for different materials, within certain limits.

## Figures and Tables

**Table 1:** Tested wires; the wires were selected due to their possible applications.

Wire	Application Examples
Copper ø 0.2	Coils, antennas, signal conductors
Copper ø 0.4	Power transmission
Constantan ø 0.2	Heating elements, sensors

**Table 2:** Grading system for the visual inspection of the embedding with the formulated criteria for each grade.

1	2	3	4	5	6
Wire embedded in full u shape	Polymer in u shape, wire not fully covered	Wire not covered, polymer higher than wire	Wire not covered, polymer lower than wire	Wire not covered but resting on polymer	Wire detached

**Table 3:** Identified parameter sets for a stable printing process for embedding wires with a diameter of 0.2 mm.

	A	B
Extrusion coefficient	0.4	0.2
Feed [mm/min]	1000	3000
Nozzle height [mm]	1	0.6
Cooling time [s]	0.25	0.075
Embedding grade	2.75	2.5
Arithmetic mean [mm]	−0.039	0.027
Standard deviation [mm]	0.197	0.267

**Figure A1:**
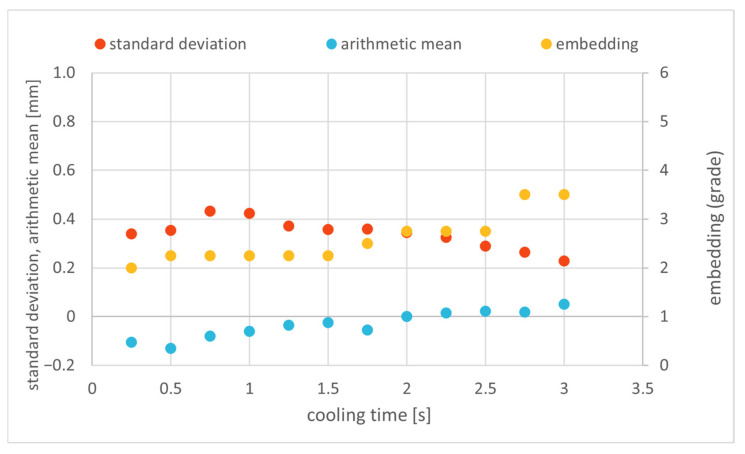
Copper wire on PLA, diameter 0.2 mm, feed 1000 mm/min, extrusion coefficient 0.8.

**Figure A2:**
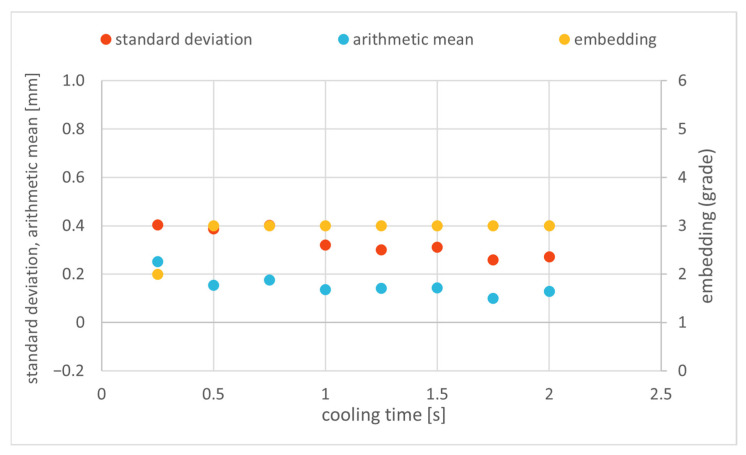
Copper wire on PLA, diameter 0.4 mm, feed 1000 mm/min, extrusion coefficient 0.4.

**Figure A3:**
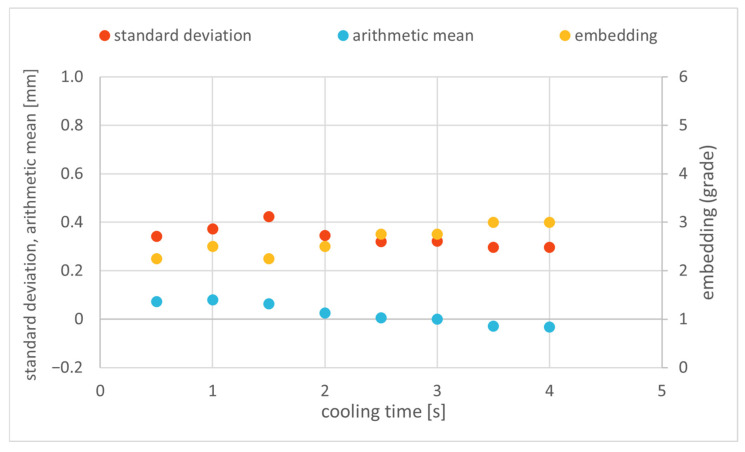
Copper wire on PLA, diameter 0.4 mm, feed 1000 mm/min, extrusion coefficient 0.8.

**Figure A4:**
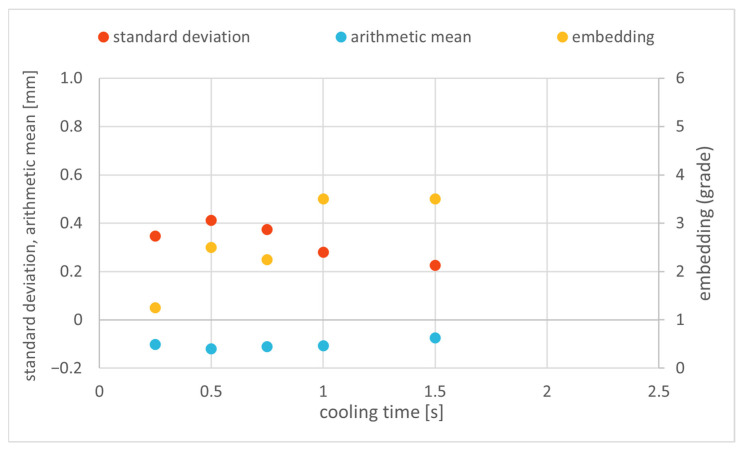
Copper wire on PETG, diameter 0.2 mm, feed 1000, extrusion coefficient 0.8.

## Data Availability

The original contributions presented in this study are included in the article. Further inquiries can be directed to the corresponding author.

## Abbreviations

AM	Additive manufacturing
CFF	Continuous Fiber Fabrication
CT	Computed tomography
IWU	Institute for Machine Tools and Forming Technology
PETG	Polyethylene terephthalate glycol
PLA	Polylactide
WEAM	Wire-encapsulating additive manufacturing

## Appendix A

The following figures show the influence of the cooling time for the correction model on the wire position (arithmetic mean, standard deviation) and embedding quality for different parameter sets.

## Appendix B

The following section shows the G-code snipped with and without the correction model. The syntax of the G-code is as follows:

G1/G3—Movement command
X/Y/Z—Linear axes
U/V—Rotary axes
W—Wire feed axis (absolute)
E—Filament feed axis (relative)
I/J—Relative coordinates of the circle center point
F—Feed speed

Without the correction, the arc can be printed with one command:
Straight before:
*G1 X-5 Y-24 U0 V0 W131.708 E19.2 F200;*
Arc:
*G3 X5 Y-24 I5 J0 U-180 V-180 W147.416 E6.283 F150;*
Straight after:
*G1 X5 Y24 U-180 V-180 W195.416 E19.2 F200.*
With the correction, the arc is divided up into multiple commands:
Straight before:
*G1 X-5 Y-24 U0 V0 W131.708 E28.8 F200;*
Opening phase:
*G3 X-4.564 Y-26.043 I5 J0 U7.165 V7.165 W133.724 E1.263 F141.2*
*G3 X-4.140 Y-26.804 I4.564 J2.043 U14.228 V14.228 W134.405 E0.523 F141.2*
*G3 X-3.729 Y-27.331 I4.140 J2.804 U21.299 V21.299 W134.800 E0.401 F141.2*
*G3 X-3.33 Y-27.730 I3.729 J3.331 U28.498 V28.498 W135.014 E0.338 F141.2*
*G3 X-2.944 Y-28.042 I3.33 J3.730 U35.984 V35.984 W135.082 E0.298 F141.2;*
Holding phase:
*G3 X5 Y-24 I2.944 J4.042 U-90.082 V-90.082 W146.083 E6.601 F141.2;*
Closing phase:
*G1 X5 Y-23.695 U-98.932 V-98.932 W146.580 E0.183 F141.2*
*G1 X5 Y-23.348 U-109.050 V-109.050 W147.144 E0.209 F141.2*
*G1 X5 Y-22.925 U-121.173 V-121.173 W147.813 E0.254 F141.2*
*G1 X5 Y-22.343 U-137.324 V-137.324 W148.685 E0.349 F141.2*
*G1 X5 Y-20.626 U-180 V-180 W150.790 E1.030 F141.2;*
Straight after:
*G1 X5 Y24 U-180 V-180 W195.416 E28.8 F200*.
